# Gut-Microbiota-Derived Butyric Acid Overload Contributes to Ileal Mucosal Barrier Damage in Late Phase of Chronic Unpredictable Mild Stress Mice

**DOI:** 10.3390/ijms252312998

**Published:** 2024-12-03

**Authors:** Chen Wang, Mei Qiu, Shuo Wang, Jinjin Luo, Ling Huang, Qi Deng, Zhijia Fang, Lijun Sun, Ravi Gooneratne

**Affiliations:** 1 Guangdong Provincial Key Laboratory of Aquatic Product Processing and Safety, College of Food Science and Technology, Guangdong Ocean University, Zhanjiang 524088, China; 18642326533@163.com (C.W.); 2112203018@stu.gdou.edu.cn (J.L.); 2112203048@stu.gdou.edu.cn (L.H.); gdoudengqi@163.com (Q.D.); fzj4437549@163.com (Z.F.); suncamt@126.com (L.S.); 2 College of Agriculture and Biotechnology, Sun Yat-sen University, Shenzhen 518107, China; wangsh569@mail2.sysu.edu.cn; 3 Department of Wine, Food and Molecular Biosciences, Faculty of Agriculture and Life Sciences, Lincoln University, P.O. Box 85084, Lincoln 7647, New Zealand; ravi.gooneratne2@lincolnuni.ac.nz

**Keywords:** chronic unpredictable mild stress, ileal mucosal barrier damage, gut microbiota, amino acid metabolism, butyric acid, periodic effects, depression behavior, inflammation, intestinal stem cell

## Abstract

Intestinal mucosal barrier damage is regarded as the critical factor through which chronic unpredictable mild stress (CUMS) leads to a variety of physical and mental health problems. However, the exact mechanism by which CUMS induces intestinal mucosal barrier damage is unclear. In this study, 14, 28, and 42 d CUMS model mice were established. The indicators related to ileal mucosal barrier damage (IMBD), the composition of the ileal microbiota and its amino acid (AA) and short-chain fatty acid (SCFA) metabolic functions, and free amino acid (FAA) and SCFA levels in the ileal lumen were measured before and after each stress period. The correlations between them are analyzed to investigate how CUMS induces intestinal mucosal barrier damage in male C57BL/6 mice. With the progression of CUMS, butyric acid (BA) levels decreased (14 and 28 d) and then increased (42 d), and IMBD progressively increased. In the late CUMS stage (42 d), the degree of IMBD is most severe and positively correlated with significantly increased BA levels (*p* < 0.05) in the ileal lumen and negatively correlated with significantly decreased FAAs, such as aspartic, glutamic, alanine, and glycine levels (*p* < 0.05). In the ileal lumen, the abundance of BA-producing bacteria (*Muribaculaceae*, *Ruminococcus*, and *Butyricicoccus*) and the gene abundance of specific AA degradation and BA production pathways and their related enzymes are significantly increased (*p* < 0.05). In addition, there is a significant decrease (*p* < 0.05) in the abundance of core bacteria (*Prevotella*, *Lactobacillus*, *Turicibacter*, *Blautia*, and *Barnesiella*) that rely on these specific AAs for growth and/or are sensitive to BA. These changes, in turn, promote further colonization of BA-producing bacteria, exacerbating the over-accumulation of BA in the ileal lumen. These results were validated by ileal microbiota in vitro culture experiments. In summary, in the late CUMS stages, IMBD is related to an excessive accumulation of BA caused by dysbiosis of the ileal microbiota and its overactive AA degradation.

## 1. Introduction

With increasing competitive societal pressure, people are continuously exposed to long-term irregular and unpredictable negative factors, including the environment, diet, and lifestyle, with the body experiencing chronic unpredictable mild stress (CUMS) [1,2]. The main manifestations of CUMS are digestive system disorders, immune dysfunction, and neuromodulation imbalance [3,4,5]. CUMS imposes many risks to human physiological and psychological health. It is revealed that up to 70% of chronic diseases are directly related to CUMS, including irritable bowel syndrome, inflammatory bowel disease, systemic inflammation, and depression [3,4,5]. However, the current interventions for CUMS-induced health problems (such as cognitive behavior therapy, use of selective serotonin reuptake inhibitors, and transcranial magnetic stimulation) have limited efficacy and are associated with multiple side effects [6,7,8]. It is now a global health problem and imposes a heavy burden on the society and economy. The pathogenesis of CUMS is still poorly understood, and an in-depth research is urgently needed.

Previous studies have suggested that the mechanism by which CUMS induces mental disease, including depression, is related to increased inflammation and impaired neurotransmitter secretion in the brain [9,10]. However, there is growing interest in the potential involvement of the intestinal barrier in stress-related diseases [11,12]. The small intestinal mucosa is responsible for digestion and absorption of nutrients and plays a crucial role in regulating the body’s endocrine, immune, and nervous system functions [2,4]. It is suggested that CUMS can markedly impair small intestinal mucosal barrier function [2,5,13], leading to increased intestinal permeability and translocation of pro-inflammatory factors, microbes, antigens, and a variety of intestinal and psychiatric disorders [14,15]. Therefore, it is essential to understand the mechanism of CUMS-induced small intestinal mucosal barrier damage to prevent and control CUMS-related diseases. However, the mechanism of how CUMS affects the small intestinal mucosal barrier and its function is currently unknown, and further studies are needed.

There have been investigations on the mechanisms of CUMS-associated impairment of the small intestinal mucosal barrier and its function. CUMS-induced increases in pro-inflammatory cytokines levels in the small intestine, impaired intestinal mucosal glutamine absorption, and reduced short-chain fatty acid (SCFA) levels can exacerbate intestinal barrier dysfunction [16,17,18]. Despite measures taken in some studies to reduce intestinal inflammation, glutamine supplementation, and increased dietary butyric acid (BA) levels, intestinal damage did not significantly improve [17,19]. These findings suggest that CUMS-induced small intestinal mucosal barrier and functional impairment may involve other mechanisms.

Normal physiological intestinal BA concentrations provide energy to intestinal mucosal cells and regulate the intestinal barrier function by promoting tight junction (TJ) protein expression and mucus production, maintaining intestinal homeostasis and thus preserving normal physiological functions of the intestine [20]. Most current studies suggest that the intestinal mucosal barrier damage caused by CUMS is associated with low BA levels in the intestinal lumen. However, some reports described high fecal BA concentrations in CUMS model mice [21]. Some patients with mental disorders such as depression and autism also exhibit high intestinal BA concentrations. Furthermore, slightly higher than physiological concentrations of BA can markedly inhibit cultured colonic epithelial stem cell proliferation [22]. Unlike colonic stem cells, ileal stem cells lack an intact crypt-protective structure and cannot secrete sufficient BA-degrading enzymes [22,23]. Thus, it can be inferred that excessive BA accumulation in the ileal lumen may be one of the crucial causes of ileal mucosal barrier damage (IMBD).

BA in the gut is mainly produced by BA-producing bacteria using specific polysaccharides and/or amino acids [24]. Most BA synthesis is closely related to the abundance of intestinal BA-producing bacteria, which is influenced by specific nutrients and other competing flora [20,25]. Tyrosine metabolism is disrupted in CUMS mice resulting in a decreased L-tyrosine in feces [17,26]. Similarly, AA dysregulation in CUMS mice can lead to decreased L-glutamine in feces [26]. These studies suggest that CUMS induce dysregulation of some AA metabolism. However, the relationship between ileal microbiota and free amino acid (FAA) levels within the gut ecological niche requires further investigation.

Significant inconsistencies are seen regarding the effect of CUMS on the intestinal mucosal barrier damage and gut microbiota composition. Some studies reported that CUMS decreased the TJ proteins occludin and ZO-1 expressions and caused significant damage to the gut [27]. Other studies have shown that CUMS did not cause significant damage to the intestinal mucosal tissue structure despite reducing the expression of intestinal TJ proteins and mucins [27,28]. There is also a significant disagreement regarding the effects of CUMS on the gut microbiota. The ratio of the bacteria Firmicutes to Bacteroides is significantly increased in CUMS model animals [29]. In contrast, another study reported a significant decrease in the ratio of Firmicutes to Bacteroides [30]. These findings indicate that there is a clear controversy in the current understanding of relevant indicators of the structure and function of the intestinal mucosal barrier in CUMS model animals and depressed patients. This controversy may be because CUMS-induced gut-related indices are dynamic processes regarding time, and significant differences in the model animals, experimental time periods and protocols are used in different studies. Therefore, it is important to investigate the changes in the structure of the intestinal mucosal barrier and gut microbiota composition in the CUMS model mice during different periods of stress to elucidate the mechanisms underlying the intestinal injury induced by CUMS.

In this study, CUMS model male mice were investigated for 14, 28, and 42 d. The physiological parameters, depression-like behavior, serum inflammatory levels, IMBD indicators, microbiota composition and function, and FAA and SCFA (including BA) concentrations in the ileal lumen were analyzed before and after each stress period. This study aims to examine the effects of CUMS on mice, focusing on the relationship between IMBD and BA over-enrichment in the ileal lumen and the correlation between ileal lumen BA over-enrichment and the composition of the ileal microbiota and its nutrient metabolism. The ultimate goal of this study is to provide a theoretical basis to reveal the possible mechanism of CUMS-induced excessive accumulation of BA in the ileal lumen and its correlation with IMBD.

## 2. Results

### 2.1. Effects of CUMS on Mice Body Weight, Food Intake, and Viscera Coefficient

**Figure 1:**
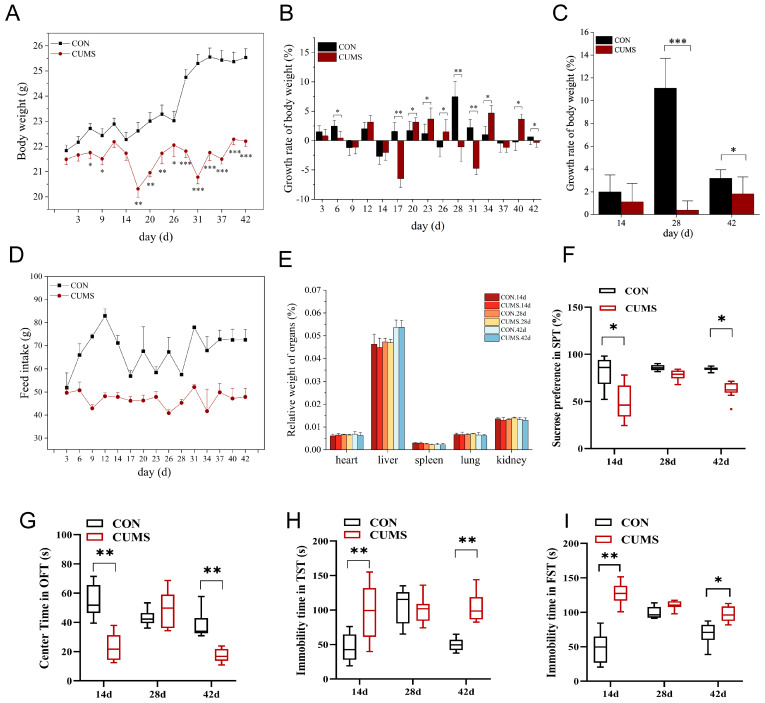
Effect of chronic unpredictable mild stress (CUMS) on physiological parameters and depression-like behavior in mice. (**A**) Body weight; (**B**) body weight growth rate per 3 d; (**C**) body weight growth rate per 14 d; (**D**) food intake; (**E**) viscera coefficient; (**F**) sucrose preference test (SPT); (**G**) time in the central area in the open field test (OFT); (**H**) immobility time in the tail suspension test (TST); (**I**) immobility time in the forced-swim test (FST). Data are shown as mean ± SEM (*n* = 15). * *p* < 0.05, ** *p* < 0.01, *** *p* < 0.001 vs. CON. Control (CON) group, chronic unpredictable mild stress (CUMS) group.

At day 6, 9 and 26, the body weight in the CUMS model group was significantly decreased (*p* < 0.05 vs. CON group). At day 17, 20, and 23, the body weight in the CUMS model group was extremely significantly decreased (*p* < 0.01 vs. CON group). At 29 d and beyond, the body weight in the CUMS model group was extremely significantly decreased (*p* < 0.001 vs. CON group). At 14 d, the body weight gain in the CUMS model group showed signs of slowing down but not significantly compared to the CON group. However, at 28 d, a highly significant reduction in the rate of body weight gain was observed in the CUMS model group compared to the CON group (*p* < 0.001). At 42 d, the rate of body weight gain in the CUMS model group continued to remain significantly lower than in the CON group (*p* < 0.05) (Figure 1A–C). The CUMS model group consumed less food at all three time periods (Figure 1D) compared to the CON group, but there was no significant effect on any of the viscera coefficients (Figure 1E). These results suggest that food intake is decreased in CUMS, and this resulted in a body weight loss.

### 2.2. Effect of CUMS on Depression Behavior of Mice

At 14 d, the sucrose preference in the sucrose preference test (SPT) (Figure 1F) and the residence time in the central region in the open field test (OFT) (Figure 1G) were significantly decreased in the CUMS model group (*p* < 0.05 vs. CON group), and the immobility time was highly significantly increased in both the tail suspension test (TST) (Figure 1H) and the forced-swim test (FST) (Figure 1I) (*p* < 0.01 vs. CON group). At 28 d, none of these four behavioral types were significant between the CUMS model group and the CON group. At 42 d, the changing trends in the four behavioral types in the CUMS model group compared to the CON group were somewhat like that at 14 d. These results indicate that in both the early and late stages of CUMS, the mice exhibited markedly depressive-like behavior, such as lack of pleasure, fear, anxiety and alertness, and a reduced desire to survive in a distressed environment, but in the middle stage of CUMS, the mice seemed to have been able to cope.

### 2.3. Effect of CUMS on the Mice IMBD

**Figure 2:**
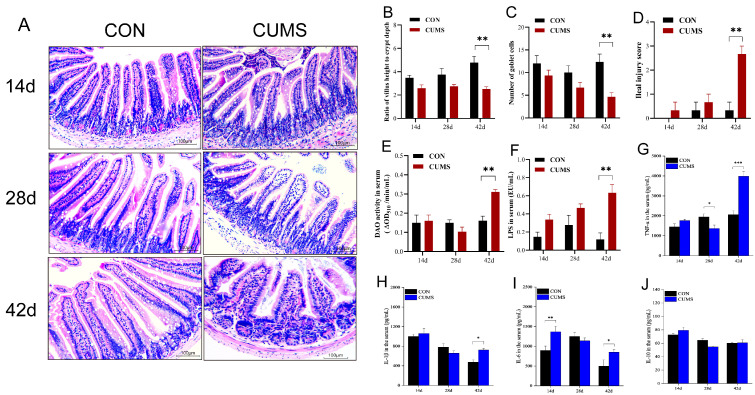
Effect of chronic unpredictable mild stress (CUMS) on ileal mucosal barrier damage (IMBD), DAO and LPS concentrations, and serum inflammatory marker levels in mice on 14, 28, and 42 d. (**A**) Histological changes in the ileal tissue (hematoxylin and eosin stain, magnification ×200); (**B**) ratio of villi height to crypt depth; (**C**) mean number of goblet cells in the visual field; (**D**) IMBD score; (**E**) Diamine Oxidase (DAO); (**F**) lipopolysaccharide (LPS); (**G**) TNF-α; (**H**) IL-1β; (**I**) IL-6; (**J**) IL-10. Data are shown as mean ± SEM (*n* = 5). * *p* < 0.05, ** *p* < 0.01, *** *p* < 0.001 vs. CON.

Histopathological changes at 14, 28, and 42 d are shown in Figure 2A (hematoxylin and eosin stain, magnification ×200). At 14 d and 28 d, the ratio of ileal villus height to crypt depth (Figure 2B) and the number of goblet cells (Figure 2C) decreased in the CUMS model group compared to the CON group, and the IMBD score (Figure 2D) and the serum LPS level increased, but none of them were significantly different. At 42 d, in the CON group, ileal villi and the crypts showed normal morphology with many goblet cells observed between the mucosal columnar cells (Figure 2A). In contrast, the ileal mucosal tissue of mice in the CUMS model group was severely damaged, characterized by goblet cell depletion, crypt hyperplasia, exposure of the lamina propria, disorganized intestinal epithelial cells, partial villi loss, and increased ileal barrier permeability. Compared with the CON group, the ratio of ileal villus height to crypt depth and the number of goblet cells in the CUMS model group were highly significantly decreased (*p* < 0.01), and the ileal injury score and the serum diamine oxidase (DAO) and lipopolysaccharide (LPS) levels (Figure 2E,F) were highly significantly increased (*p* < 0.01). These results suggest that there was no damage to ileal tissue or effect on ileal barrier permeability during the initial and intermediate stages of CUMS, but during the late stages of CUMS, the ileal mucosal tissue undergoes significant damage, leading to a marked increase in the ileal mucosal tissue injury score.

### 2.4. Effect of CUMS on Inflammatory Markers Levels in the Serum of Mice

The serum concentrations of the pro-inflammatory factors (TNF-α, IL-1β, IL-6) are shown in Figure 2G–I. At 14 d, the changes in the TNF-α and IL-1β levels were not significant in the CUMS model group, but the level of IL-6 was highly significantly increased (*p* < 0.01 vs. CON group). At 28 d, the level of TNF-α was significantly lower in the CUMS model group (*p* < 0.05 vs. CON group), but the changes in the IL-1β and IL-6 levels were not significant. At 42 d, the levels of TNF-α, IL-1β, and IL-6 were all significantly increased in the CUMS model group compared to the CON group (*p* < 0.05), with a significant increase of 93.63% in the level of TNF-α. But none of the serum levels of the anti-inflammatory factor IL-10 (Figure 2J) were significantly affected by CUMS. The fluctuations in serum concentrations of TNF-α, IL-1β, and IL-6 at 14, 28, and 42 d reflect a dynamic inflammatory response in the CUMS model, shedding light on the progression of stress-induced inflammatory states. IL-6 may be an early biomarker of acute inflammatory response to CUMS and play a key role in the initial phase of inflammatory response. The reduced TNF-α levels at 28 d potentially indicate a compensatory mechanism where prolonged exposure to stress may temporarily downregulate certain pro-inflammatory responses to mitigate tissue damage. However, by 42 d, the marked increase in TNF-α, IL-1β, and IL-6 indicate an escalation in systemic inflammation. This later-phase elevation may reflect an overwhelmed compensatory response. It shows that CUMS eventually induces sustained inflammation. The consistent, non-significant effect on IL-10 levels suggests that CUMS may primarily disrupt pro-inflammatory rather than anti-inflammatory pathways. CUMS exposure may impair the body’s ability to initiate a sufficient anti-inflammatory response, further exacerbating an inflammatory state.

### 2.5. Effect of CUMS on Ileal Occludin, Zonula Occludens-1 (ZO-1), Mucoprotein 2 (Muc2), and Olfactomedin 4 (Olfm4+) Protein Expressions in Mice

**Figure 3:**
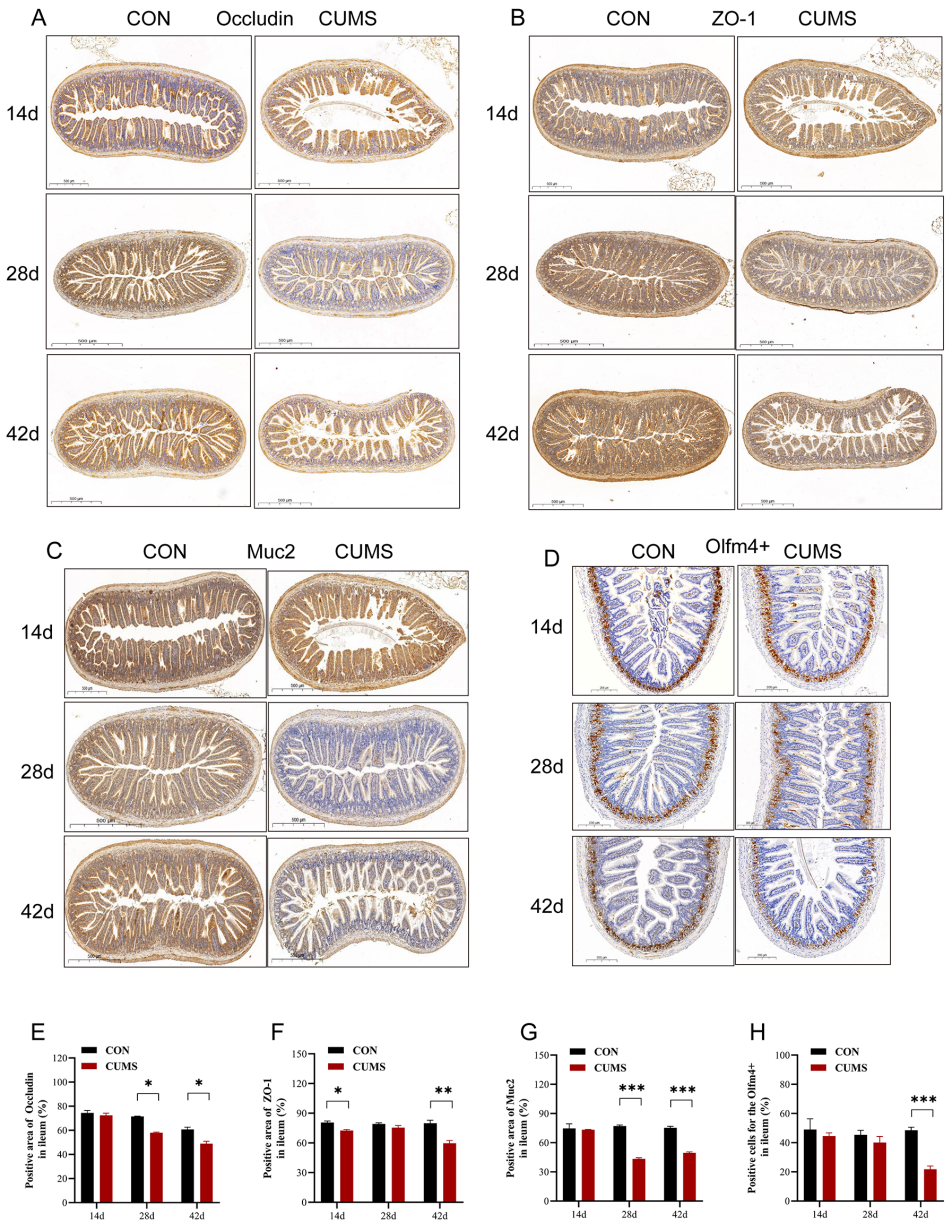
Effect of chronic unpredictable mild stress (CUMS) on ileal occludin, ZO-1, Muc2, and Olfactomedin 4 (Olfm4+) protein expression in mice. (**A**–**D**) Immunohistochemistry of occludin, ZO-1, Muc2, and Olfm4+ proteins in the ileal tissue in different groups; (**E**) positive areas of occludin in the ileum; (**F**) positive areas of ZO-1 in the ileum; (**G**) positive areas of Muc2 in the ileum; (**H**) positive cells for Olfm4+ in the ileum. Data are shown as mean ± SEM (*n* = 3). * *p* < 0.05, ** *p* < 0.01, *** *p* < 0.001 vs. CON.

The immunohistochemistry of occludin, ZO-1, Muc2, and Olfm4+ proteins is shown in Figure 3A–D. At 14 d, only the ileal TJ protein ZO-1 expression was significantly decreased in the CUMS group (*p* < 0.05 vs. CON group) (Figure 3F) with no significant changes in the expression of either the TJ proteins occludin and Muc2 (Figure 3E,G). At 28 d, there was no significant change in the expression of ileal ZO-1 in the CUMS model group compared to the CON group, but significant decreases in the expression of occludin and Muc2 were observed (*p* < 0.05). At 42 d, the expression of occludin, ZO-1, and Muc2 in the ileal tissues of the CUMS model group was significantly decreased compared to the CON group (*p* < 0.05).

At 14 d and 28 d, there was no significant change in the ratio of positive cells for Olfm4+ in the ileum of the CUMS model group compared to the CON group (Figure 3H). At 42 d, the ratio of Olfm4+ positive cells in the ileum was highly significantly decreased in the CUMS model group compared to the CON group (*p* < 0.001).

### 2.6. Effect of CUMS on the Composition and Function of Ileal Gut Microbiota in Mice

#### 2.6.1. Diversity of the Ileal Gut Microbiota

**Figure 4:**
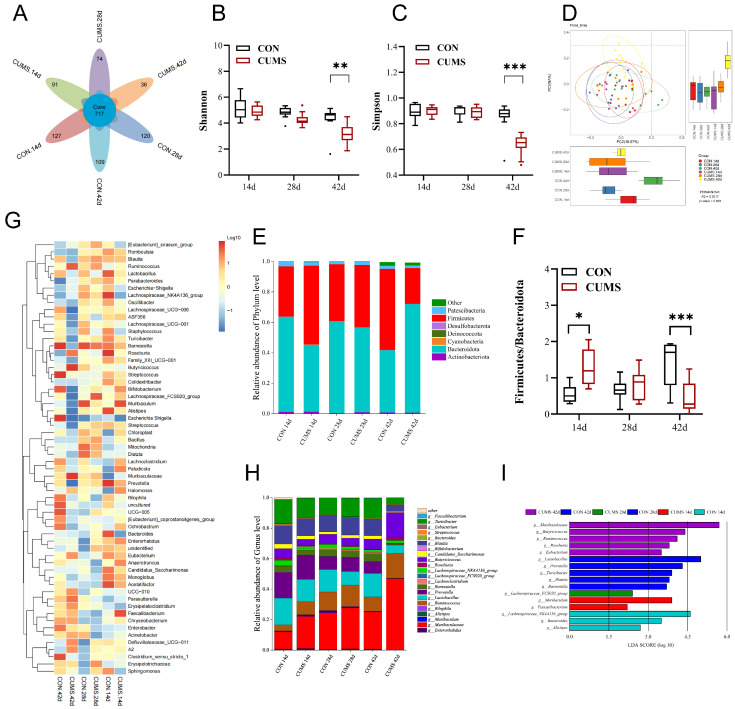
Effect of chronic unpredictable mild stress (CUMS) on the diversity and composition of ileal gut microbiota in mice. (**A**) Venn diagram based on OTU; (**B**) Shannon’s index; (**C**) Simpson’s index; (**D**) principal coordinate analysis (PCoA) of gut microbiota. Gut microbiota changes at the (**E**) phylum and (**H**) genus levels. (**F**) Ratio of Firmicutes and Bacteroidota between experimental groups. (**G**) Horizontal clustering heat map of the gut microbiota. (**I**) Classification of unit groups based on LDA histograms for taxa showed a significant difference between groups (*p* < 0.05). Data are expressed as mean ± SEM (*n* = 10). * *p* < 0.05, ** *p* < 0.01, *** *p* < 0.001 vs. CON.

During all three time periods, the number of OTUs in the CUMS model group decreased compared to the CON group. OTUs decreased from 844 to 808 at 14 d, from 837 to 791 OTUs at 28 d, and from 826 to 753 OTUs at 42 d, with the highest decrease at 42 d (Figure 4A). At 14 d and 28 d, there was no significant effect of CUMS on the α-diversity and β-diversity of the gut microbiota compared to the CON group, but at 42 d, there was a highly significant decrease in α-diversity, including Shannon and Simpson, in the CUMS model group (*p* < 0.01 vs. CON group). The confidence ellipses of the CON and CUMS groups differed significantly from one another, meaning that there was a significant difference in β-diversity (Figure 4B–D).

#### 2.6.2. Composition of the Ileal Gut Microbiota in Mice

The ileal microbiota was analyzed at two taxonomic levels. At the phylum level (Figure 4E,F), Firmicutes and Bacteroidota made up >90% of the total flora. At 14 d, compared to the CON group, the ratio of Firmicutes to Bacteroidota was highly significantly higher in the CUMS model group (*p* < 0.05). At 28 d, there was no significant difference in the ratio of Firmicutes to Bacteroidota in the CUMS model group (vs. CON group). At 42 d, the ratio of Firmicutes to Bacteroidota was highly significantly lower in the CUMS model group (*p* < 0.001 vs. CON group).

At 42 d, the most significant differences in genus composition between the CON and CUMS groups are shown in the clustered heat map (Figure 4G). *Muribaculaceae*, *Ruminococcus*, *Butyricicoccus*, *Lactobacillus,* and *Prevotella* were the predominant genera within the gut microbiota of each group (Figure 4H). To identify species exhibiting statistically significant differences between each time period group, a LEfSe analysis was conducted, which involved 16 characteristic marker bacteria obtained in the CUMS groups that were quite distinct from the CON group in three periods (Figure 4I).

**Figure 5:**
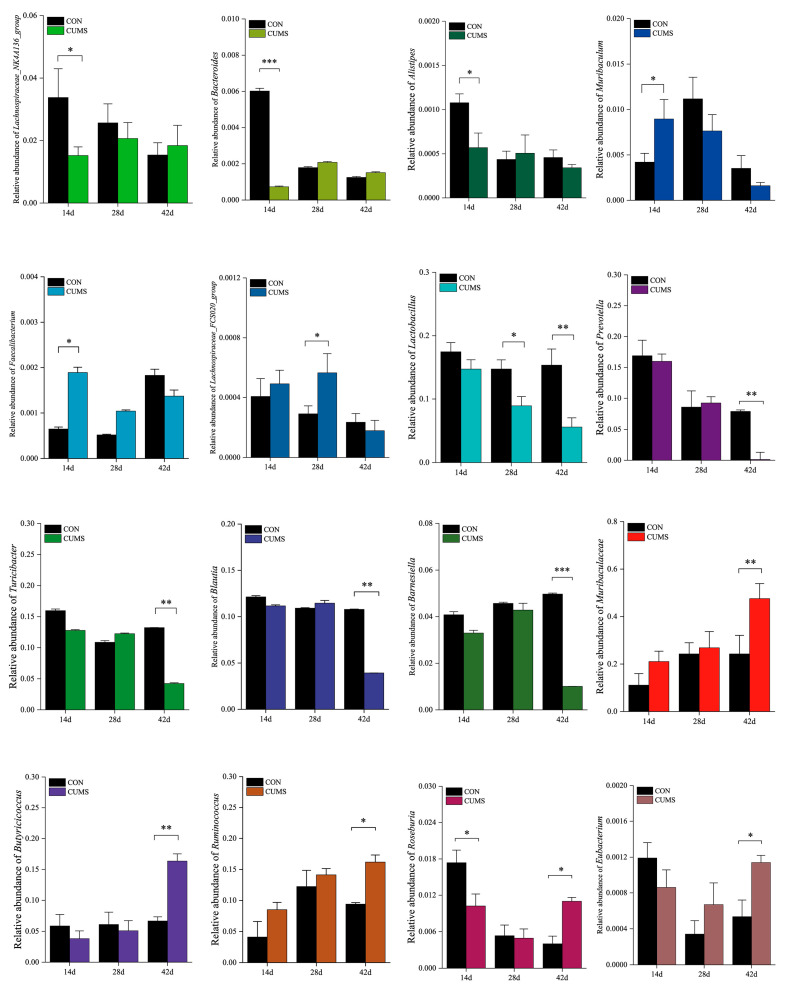
Effects of chronic unpredictable mild stress (CUMS) on the abundance of 16 characteristic marker bacteria in the ileal microbiota in mice. Data are expressed as mean ± SEM (*n* = 10). * *p* < 0.05, ** *p* < 0.01, *** *p* < 0.001 vs. CON.

As illustrated in Figure 5, at 14 d, the relative abundance of the CUMS model groups *Lachnospiraceae_NK4A136_group*, *Bacteroides*, *Alistipes*, and *Roseburia* were significantly decreased (*p* < 0.05) compared to the CON group, and the relative abundances of *Muribaculum* and *Faecalibacterium* were significantly increased (*p* < 0.05). At 28 d, the relative abundance of the *Lachnospiraceae_FCS020_group* was significantly increased (*p* < 0.05) and of *Lactobacillus* significantly decreased (*p* < 0.05) in the CUMS model group compared to the CON group. At 42 d, the relative abundances of *Lactobacillus*, *Prevotella*, *Turicibacter*, *Blautia*, and *Barnesiella* in the CUMS model group were significantly lower (*p* < 0.05) compared to the CON group, and the relative abundances of *Muribaculaceae*, *Butyricicoccus*, *Ruminococcus*, *Roseburia*, and *Eubacterium* were significantly increased (*p* < 0.05). These results show that in contrast to the ileal-microbiota-changes trend in the CON group, the relative abundance of *Muribaculum*, *Lactobacillus*, and *Prevotella* gradually decreased over time in the CUMS model group, while the relative abundance of *Muribaculaceae*, *Butyricicoccus*, and *Ruminococcus* gradually increased.

#### 2.6.3. Predictive Analysis of Ileal Microbiota Metabolism

**Figure 6:**
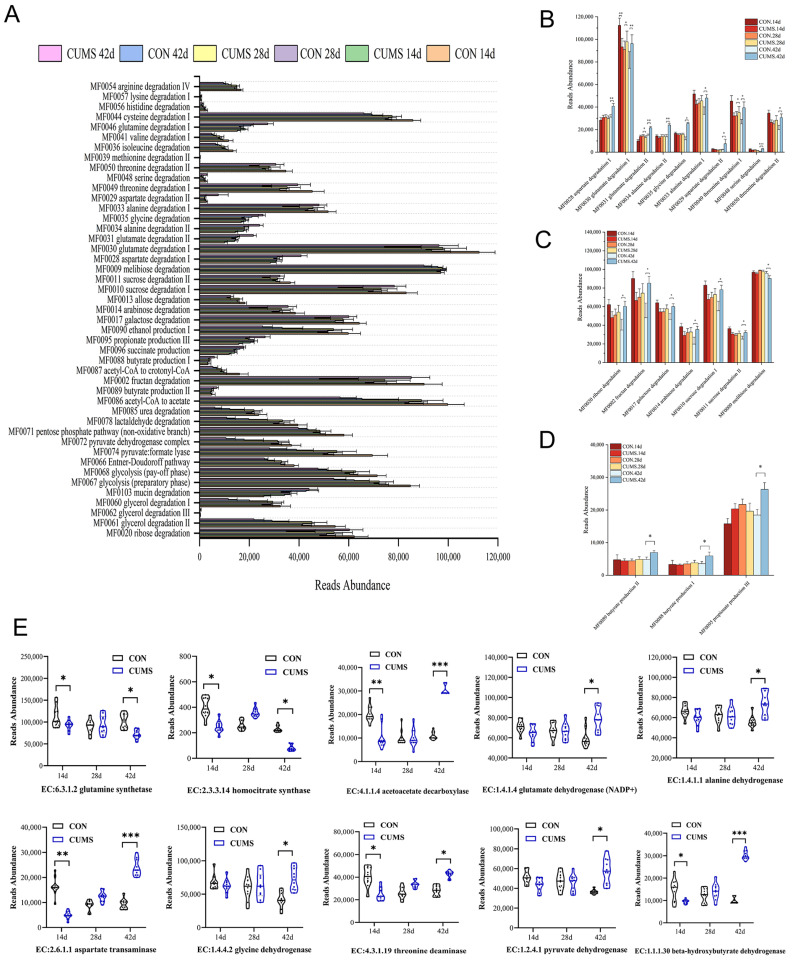
Effects of chronic unpredictable mild stress (CUMS) on the gene abundance of the ileal microbiota AAs, sugars, and SCFAs metabolic pathways and related enzymes in mice. (**A**) Analysis of the metabolic prediction of the Gut Metabolic Module (GMM) module of the gut microbiota, (**B**) changes in gene abundance of amino acid (AA) metabolism pathway, (**C**) changes in gene abundance of sugar metabolism pathway, (**D**) changes in gene abundance of SCFA metabolism, (**E**) dynamic variations in gene abundance of AA degradation and butyric acid (BA) production metabolism-related enzymes. Data are expressed as mean ± SEM (*n* = 10). * *p* < 0.05, ** *p* < 0.01, *** *p* < 0.001 vs. CON.

Predictive analyses of the metabolic function of the ileal microbiota based on the enrichment of metabolic function genes in each group of gut flora revealed that the gene abundances in metabolic pathways for AAs, sugars, and SCFAs exhibited the most significant differences in the ileal microbiota (Figure 6A). In particular, AA and sugar metabolism genes were enriched in degradation metabolism, while SCFAs metabolism genes were mainly enriched in production metabolism. The difference between the CON and CUMS groups was significantly higher at 42 d than at 14 and 28 d. The gene abundances of the aspartate, glutamate, alanine, glycine, threonine and serine degradation pathways (Figure 6B), the ribose, fructan, galactose, arabinose and sucrose degradation pathways (Figure 6C), and the butyrate and propionate production pathways were significantly increased in the 42 d CUMS group (*p* < 0.05) compared with the 42 d CON group (Figure 6D).

In the gene abundance analyses of enzymes in the AA, sugar, and SCFA metabolic pathways, ten enzymes were significantly different between the CON and CUMS groups at the three time periods (Figure 6E) and were most pronounced in the AA degradation and BA production pathways. At 14 d, the gene abundances of AA-degrading enzymes (aspartate transaminase [EC 2.6.1.1], threonine deaminase [EC 4.3.1.19]) and BA-producing enzymes (acetoacetate decarboxylase [EC 4.1.1.4] and beta-hydroxybutyrate dehydrogenase [EC 1.1.1.30]) were significantly decreased in the CUMS model group compared to the CON group (*p* < 0.05). At 28 d, there were no significant differences in gene abundance of enzymes in either the AA degradation or the BA production metabolic pathways in the CUMS model group compared to the CON group. At 42 d, the gene abundances of AA-degrading enzymes (aspartate transaminase [EC 2.6.1.1], threonine deaminase [EC 4.3.1.19], glutamate dehydrogenase [EC 1.4.1.4], alanine dehydrogenase [EC 1.4.1.1], glycine dehydrogenase [EC 1.4.4.2]) and BA-producing enzymes (pyruvate dehydrogenase [EC 1.2.4.1], acetoacetate decarboxylase [EC 4.1.1.4], beta-hydroxybutyrate dehydrogenase [EC 1.1.1.30]) were significantly increased in the CUMS group (*p* < 0.05) compared to the CON group.

### 2.7. Effects of CUMS on FAAs and SCFAs Levels in the Ileal Contents of Mice

**Figure 7:**
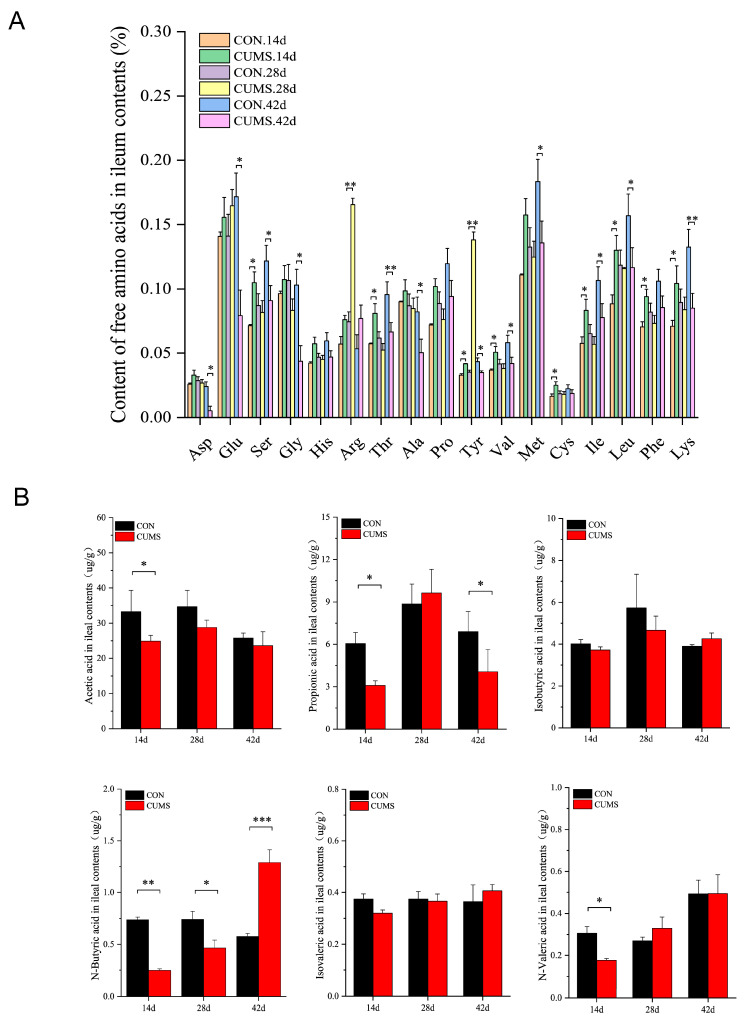
Effects of chronic unpredictable mild stress (CUMS) on free amino acid (FAA) and short-chain fatty acid (SCFA) levels in the mice ileal contents. (**A**) Mice FAAs levels in the ileal contents, (**B**) mice SCFA (acetic, propionic, isobutyric, N-butyric, isovaleric, N-valeric) concentrations in the ileal contents. Data are expressed as mean ± SEM (*n* = 6). * *p* < 0.05, ** *p* < 0.01, *** *p* < 0.001 vs. CON.

Further examination of the levels of FAAs and SCFAs in the ileal lumen showed that at 14 d, the levels of serine (Ser), threonine (Thr), tyrosine (Tyr), valine (Val), cysteine (Cys), isoleucine (Ile), leucine (Leu), phenylalanine (Phe), and lysine (Lys) were significantly increased in the CUMS model group compared to the CON group (*p* < 0.05). At 28 d, the levels of argnine (Arg) and Tyr were significantly increased in the CUMS model group (*p* < 0.05 vs. CON group), and the other AAs showed a decreasing trend, but the differences were not significant. At 42 d, the levels of aspartic acid (Asp), glutamic acid (Glu), alanine (Ala), glycine (Gly), methionine (Met), Ser, Thr, Tyr, Val, Ile, Leu, and Lys were significantly decreased in the CUMS model group compared to the CON group (*p* < 0.05) (Figure 7A).

At 14 d, the acetic, propionic, butyric, and valeric acid levels were significantly decreased in the CUMS model group compared to the CON group (*p* < 0.05) (Figure 7B). At 28 d, the BA level was significantly reduced in the CUMS model group (*p* < 0.05) vs the CON group. At 42 d, the propionic acid level was significantly decreased (*p* < 0.05), and the BA level was highly significantly increased (*p* < 0.001) in the CUMS model group compared to the CON group.

### 2.8. Correlation Analysis

#### 2.8.1. Correlation Analysis of FAAs, BA, Gut Key Marker Bacteria, and Gene Abundance of Predicted Metabolic Pathways and Associated Enzymes

**Figure 8:**
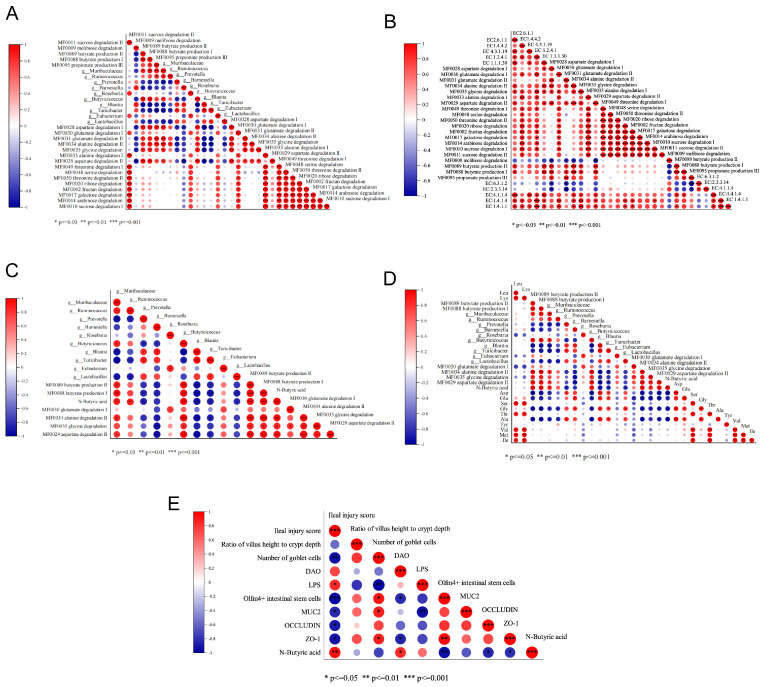
Correlation analysis of the ileal content levels of free amino acid (FAA) and butyric acid (BA); the abundance of the key ileal marker bacteria; and the gene abundance of amino acid (AA), sugar, and short-chain fatty acid (SCFA) metabolic pathways and associated enzymes in the ileal microbiota. And correlation analysis of the BA levels in the ileal contents and ileal mucosal barrier indices. (**A**) Correlation between the key ileal marker bacteria and gene abundance of ileal microbiota AA, sugar, and SCFA metabolism. (**B**) Correlation between the gene abundance of ileal microbiota AA, sugar, and SCFA metabolism and the AA-degradation- and BA-production-metabolism-related enzymes. (**C**) Relationship between the key ileal gut marker bacteria, BA, and gene abundance of AA degradation and BA production metabolism. (**D**) Relationship between the key ileal marker bacteria, BA, FAAs, and gene abundance of AA degradation and BA production metabolism. (**E**) Correlation analysis of the BA levels in the ileal contents and ileal mucosal barrier indices. * *p* < 0.05, ** *p* < 0.01, *** *p* < 0.001. Leucine (Leu), lysine (Lys), aspartic acid (Asp), glutamic acid (Glu), serine (Ser), glycine (Gly), threonine (Thr), alanine (Ala), tyrosine (Tyr), valine (Val), methionine (Met), isoleucine (Ile), diamine oxidase (DAO), lipopolysaccharide (LPS), Zonula occludens-1 (ZO-1), Mucoprotein 2 (Muc2), and Olfactomedin 4 (Olfm4+).

To determine possible intrinsic associations between the ileal contents (FAA, BA levels); the abundance of the key ileal gut marker bacteria; and the gene abundance of AA, sugar, and SCFA metabolic pathways and associated enzymes in the ileal microbiota in the 42 d CUMS model group, a correlation analysis between these indicators was conducted using a Pearson’s correlation analysis (Figure 8A–D).

Firstly, the abundance of ten key ileal gut marker bacteria in the 42 d CUMS was correlated with the gene abundance of twenty metabolic pathways (AA, sugar, SCFA) in the ileal microbiota that differed significantly between groups (Figure 8A). *Muribaculaceae* positively correlated with the MF0089 butyrate production II, MF0088 butyrate production I, MF0095 propionate production III, MF0028 aspartate degradation I, MF0031 glutamate degradation II, MF0034 alaninate degradation II, MF0035 glycine degradation, and MF0029 aspartate degradation II (*p* < 0.05). *Ruminococcus* positively correlated with MF0095 propionate production III and MF0034 alanine degradation II (*p* < 0.05). *Prevotella* negatively correlated with MF0088 butyrate production I, MF0028 aspartate degradation I, MF0031 glutamate degradation II, MF0034 alanine degradation II, and MF0035 glycine degradation (*p* < 0.05). *Barnesiella* negatively correlated with MF0089 butyrate production II, MF0088 butyrate production I, MF0028 aspartate degradation I, and MF0029 aspartate degradation II (*p* < 0.05). *Butyricicoccus* positively correlated with MF0089 butyrate production II, MF0088 butyrate production I, MF0028 aspartate degradation I, MF0031 glutamate degradation II, MF0034 alanine degradation II, MF0035 glycine degradation, and MF0029 aspartate degradation II (*p* < 0.05). *Blautia* and *Turicibacter* negatively correlated with MF0089 butyrate production II, MF0088 butyrate production I, MF0095 propionate production III, MF0028 aspartate degradation I, MF0031 glutamate degradation II, MF0034 alanine degradation II, MF0035 glycine degradation, and MF0029 aspartate degradation II (*p* < 0.05). *Lactobacillus* negatively correlated with MF0088 butyrate production I and MF0034 alanine degradation II (*p* < 0.05). This suggests that in the context of 42 d CUMS, the metabolic pathways that strongly correlated with key ileal gut marker bacteria were the aspartate, glutamate, alanine, and glycine degradation metabolisms and the propionate and butyrate production metabolisms.

The gene abundance of twenty metabolic pathways (AA, sugar, SCFA) in the ileal microbiota that differed significantly (*p* < 0.05) between groups was correlated with the gene abundance of ten enzymes that differed significantly (*p* < 0.05) between groups (Figure 8B). MF0088 butyrate production I and MF0089 butyrate production II positively correlated with EC:4.1.1.4 acetoacetate decarboxylase and EC 1.1.1.30 beta-hydroxybutyrate dehydrogenase (*p* < 0.05). MF0030 glutamate degradation I positively correlated with EC:1.4.1.4 glutamate dehydrogenase (NADP+) (*p* < 0.05). MF0029 aspartate degradation II positively correlated with EC2.6.1.1 aspartate transaminase (*p* < 0.05). MF0034 alanine degradation II positively correlated with EC 1.4.1.1 alanine dehydrogenase (*p* < 0.05). MF0035 glycine degradation positively correlated with EC 1.4.4.2 glycine dehydrogenase (*p* < 0.05). This further suggests that in the context of 42 d CUMS, MF0088 butyrate production I, MF0089 butyrate production II, MF0030 glutamate degradation I, MF0029 aspartate degradation II, MF0034 alanine degradation II, and MF0035 glycine degradation pathways play a key role.

Next, gene abundance of the six metabolic pathways mentioned above was then correlated with the butyrate level and the abundance of the ten key ileal marker bacteria (Figure 8C). BA positively correlated with *Muribaculaceae*, *Ruminococcus*, *Butyricicoccus*, MF0089 butyrate production II, MF0088 butyrate production I, MF0034 alanine degradation II, MF0035 glycine degradation, and MF0029 aspartate degradation II (*p* < 0.05) and negatively correlated with *Prevotella*, *Blautia*, *Turicibacter*, and *Lactobacillus* (*p* < 0.05). This suggests that in the context of 42 d CUMS, *Muribaculaceae*, *Ruminococcus*, *Butyricicoccus*, *Prevotella*, *Blautia*, *Turicibacter*, *Lactobacillus*, and specific amino acid degradation metabolism and BA production metabolism play a key role in the process of BA overload in the ileal lumen.

Finally, the levels of the twelve FAAs that were significantly (*p* < 0.05) reduced in the 42 d CUMS model group were then correlated with the gene abundance of the six metabolic pathways mentioned above, BA levels, and the abundance of the ten key ileal marker bacteria (Figure 8D). Asp, Glu, Gly, and Ala negatively correlated with *Muribaculaceae*, *Ruminococcus*, *Butyricicoccus*, MF0089 butyrate production II, MF0088 butyrate production I, and BA (*p* < 0.05). Asp negatively correlated with MF0029 aspartic acid degradation II (*p* < 0.05). Glu negatively correlated with MF0030 glutamic acid degradation I (*p* < 0.05). Gly negatively correlated with MF0035 glycine degradation I (*p* < 0.05). Ala negatively correlated with MF0034 alanine degradation II (*p* < 0.05). In addition, Asp, Ala, Glu, and Gly positively correlated with *Prevotella*, *Barnesiella*, *Blautia*, *Lactobacillus,* and *Turicibacter* (*p* < 0.05). This suggests that in the context of 42 d CUMS, specific AAs levels in the ileal lumen also play a key role in the process of BA overload in the ileal lumen.

#### 2.8.2. Correlation Analysis of BA and IMBD Indicators

To determine possible intrinsic associations between BA levels in the ileal contents and ileal mucosal barrier indices in the 42 d CUMS model group, a correlation analysis between these indicators was conducted using the Pearson’s correlation analysis (Figure 8E). BA positively correlated with the ileal injury score and DAO and negatively correlated with the number of ileal stem cells Olfm4+ and the expression of the TJ proteins (occluding, ZO-1). These results suggest a significant positive correlation between excessively elevated BA levels in the ileal contents and the degree of IMBD in the 42 d CUMS model group.

### 2.9. 42 d Ileal Flora In Vitro Culture

#### 2.9.1. Growth Curves, pH and BA Levels, and FAA Consumption Rates in Ileal Flora In Vitro Culture

**Figure 9:**
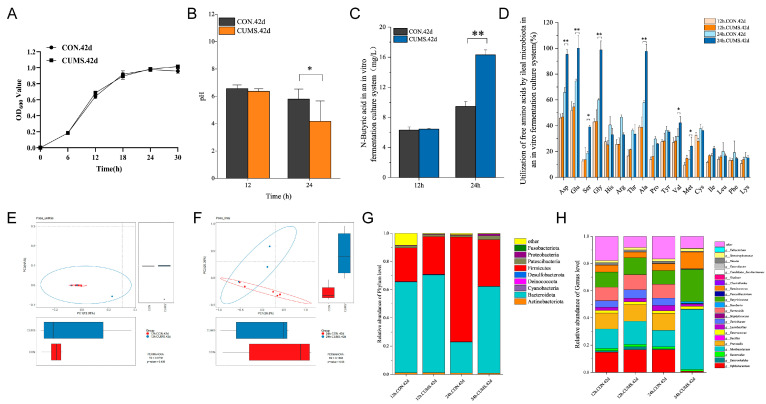
Growth curves, pH, butyric acid (BA) levels, free amino acid (FAA) consumption rates, and the composition of 42 d CON and CUMS ileal flora in vitro culture. (**A**) Growth curves, (**B**) pH, (**C**) BA levels, (**D**) FAAs consumption rates. PCoA analysis of CON 42 d and CUMS 42 d ileal flora in vitro culture at (**E**) 12 and (**F**) 24 h. Ileal microbiota in vitro culture changes at the phylum (**G**) and genus (**H**) levels. Data are expressed as mean ± SEM (*n* = 6). * *p* < 0.05, ** *p* < 0.01 vs. CON.

In the ileal microbiota in vitro cultures, the ileal flora growth is shown in Figure 9A. The rapid growth period of the microbiota was 6–18 h; 18–24 h was the stabilized growth period of the microbiota, peaking at 24 h. After a 12 h culture of the ileal microbiota in the 42 d CUMS model group, the pH and BA levels and FAAs consumption rates recovered to a level comparable to the CON group (Figure 9B–D). But at 24 h, the CUMS 42 d group significantly decreased in pH (*p* < 0.05), BA levels significantly increased (*p* < 0.01), and the rate of consumption of Asp, Glu, Ser, Gly, Ala, Val, and Met significantly increased (*p* < 0.05), compared to the CON 42 d group.

#### 2.9.2. Analysis of the In Vitro Culture Ileal Flora Composition

There was no significant difference in β-diversity between the 12 h CON 42 d and 12 h CUMS 42 d groups (Figure 9E). However, the 24 h CON 42 d group and 24 h CUMS 42 d group confidence ellipses differed significantly from one another, meaning that there was a significant difference in β-diversity (Figure 9F). At the phylum level (Figure 9G), Firmicutes and Bacteroidota made up >90% of the total flora. There was no significant difference in the relative abundance of different phyla between the 12 h CON 42 d and 12 h CUMS 42 d groups. However, the relative abundance of the Firmicutes was significantly decreased (*p* < 0.05), and the relative abundance of the Bacteroidota was significantly increased (*p* < 0.05) in the 24 h CUMS 42 d group compared with the 24 h CON 42 d group. At the genus level (Figure 9H), the abundance of bacteria that rely on specific AAs for growth, including *Prevotella,* increased, while the abundance of SCFA-producing bacteria, including *Muribaculaceae*, decreased after a 12 h culture of the ileal microbiota in the 42 d CUMS model group, recovering to a level comparable to the CON group. There was no significant difference in the relative abundance of the different genera between the 12 h CON 42 d and 12 h CUMS 42 d groups. However, following a 24 h culture, the structure of the bacterial population in the culture solution became unbalanced, with characteristics that matched the changes in the bacterial population in the ileal contents in vivo. In the 24 h CUMS 42 d group, the relative abundance of *Bifidobacterium*, *Lactobacillus*, *Prevotella*, *Barnesiella*, *Turicibacter*, *Blautia*, *Bacteroides*, and *Enterorhabdus* was highly significantly decreased (*p* < 0.001), and the relative abundance of *Muribaculaceae*, *Butyricicoccus*, *Ruminococcus*, *Roseburia*, *Faecalibacterium*, and *Eubacterium* was highly significantly increased (*p* < 0. 001) compared to the 24 h CON 42 d group.

## 3. Discussion

Intestinal mucosal barrier damage is regarded as the key mechanism by which CUMS can lead to a variety of physical and mental health problems. The exact mechanism by which CUMS induces intestinal mucosal barrier damage is unclear.

Previous studies on CUMS have, in most instances, used a single modeling time period, of two to five weeks or even longer [31,32,33,34]. This discrepancy has led to significant inconsistencies in assessing the effects of CUMS on the pathophysiological indices. Some studies found significantly different changes in the types and levels of characteristic inflammatory markers in CUMS [35,36,37]. Many studies reported that CUMS experimental procedure successfully induced depression-like behavior in mice [38,39]. However, other studies have shown that CUMS can promote behavioral adaptations [40,41]. Additionally, there are significant differences in the effects of CUMS on indicators related to IMBD. Some studies reported that CUMS decreased the TJ proteins occludin and ZO-1 expressions in the colon [27]. Others have reported that CUMS reduced mucin expression in the rat colon without causing significant damage to the histological structure of the mucosa [28]. Such observations pose a substantial challenge to exploring the mechanism of CUMS-induced body damage over different time frames. Therefore, this study used multiple sampling time points (14, 28, 42 d) to investigate the CUMS model mice.

At each time period, different key change indicators showed changes. At early stages (14 d), the mice show apparent depression-like behavior, with a significant increase (*p* < 0.01) in serum IL-6 levels and a significant decrease (*p* < 0.05) in ileal mucosal TJ protein ZO-1 expression, but the ileal microscopic changes were minimal. At 28 d, only a significant (*p* < 0.05) decrease in serum TNF-α levels and the ileal occludin and MUC2 expression was evident, and other indices, including the depression-like behavior, are no longer obvious. At 42 d, compared with the CON group, all indicators except organ coefficients and serum IL-10 levels show significant differences, with the depression-like behavior and IMBD most evident. Such outcomes suggest that the body responds to CUMS change with time. During the early to late stages of CUMS, the serum inflammatory levels and depression-like behavior fluctuated from high to low to high. In contrast, the IMBD progressively increases, which has rarely been previously reported. Due to a robust environmental adaptation and self-regulation ability [42], the body can repair the adverse effects of CUMS through self-regulation within a relatively short period. Possibly, the animals exhibited some but minimal adaptation to the stress protocol by 28 d, reflected by the physiological indexes returning to normal during the middle stage of CUMS (28 d). However, extending the stress process to 42 days may have surpassed the capacity of these adaptation mechanisms. It becomes unsustainable for the body to self-regulate, and considerable damage was evident at 42 d of CUMS, especially the IMBD. Such fluctuations may be a common phenomenon of stress. However, this phenomenon’s underlying cause(s) require further exploration. It was apparent that 28 to 42 d was the critical period for CUMS in this study for maximal damage, including to the intestinal barrier. In general, the novel findings of this study regarding the periodic response to CUMS are important reference points for understanding the mechanisms of CUMS-induced depression and other tissue damage so that effective prevention and treatment measures can be explored.

Both psychological and physiological stressors can affect gut function and trigger barrier dysfunction [43,44]. CUMS-induced increases in pro-inflammatory cytokines levels in the intestine, impaired intestinal mucosal glutamine absorption, and reduced SCFA levels, all of which can exacerbate intestinal barrier dysfunction [16,17,18]. These studies suggest that CUMS causes impairment of the intestinal mucosal barrier and its function through multiple pathways. However, these studies mainly explored the relationship between the microenvironment and the structure of the intestinal mucosal barrier. They did not reveal the mechanisms by which CUMS induces IMBD at the initial stage of intestinal mucosal development. In this study, the proliferation of Olfm4+ intestinal stem cells (ISCs) in the ileal crypt is inhibited in the 42 d CUMS model group compared with the CON, highly consistent with the manifestation of IMBD. ISCs in the intestinal mucosal crypts control intestinal mucosal epithelial renewal and damage repair through proliferation and differentiation [13,45], essential for maintaining the integrity of the intestinal mucosal barrier. Therefore, inhibition of ISC proliferation and differentiation may be the underlying cause of CUMS-induced IMBD. However, the specific mechanism by which CUMS inhibits the proliferation and differentiation of Olfm4+ ISCs is unknown and requires further investigation.

Physiological concentrations of BA are a primary energy source and a functional regulator in the intestinal mucosa and are essential for repairing intestinal mucosal barrier damage [20]. BA levels are generally reduced in the feces of CUMS model mice, and increased BA levels help to maintain intestinal homeostasis [46,47]. In this study, BA levels in the ileal lumen of CUMS mice were initially decreased and then gradually increased, especially in the 42 d CUMS model group, where BA levels are over-enriched well above the normal physiological concentration observed in the CON group. This process was accompanied by a significant decrease (*p* < 0.001) in the number of ISCs in the ileal crypt with significant damage to the mucosa. Significantly increased BA levels positively correlated with the ileal injury score and DAO and negatively correlated with the number of ileal stem cells (Olfm4+). Based on these findings, it is speculated that excessive BA may inhibit ileal ISC proliferation, which triggers IMBD.

The normal function of intestinal epithelial cells depends on the normal proliferation and differentiation of ISCs at the bottom of the crypt [13,45]. Notably, BA at slightly higher than physiological concentrations significantly inhibits the growth and differentiation of intestinal organoids cultivated from isolated, cultured colonic crypt stem cells. In particular, when colonic stem cells (due to crypt damage or naturally crypt-less organisms such as zebrafish) are exposed to intestinal luminal BA, BA strongly inhibits their proliferation, leading to crypt developmental disorders and delayed wound repair [22]. Such clinical outcomes suggest excess BA may cause colonic mucosal damage under certain conditions. However, under normal physiological conditions, BA does not cause damage to the colonic mucosal barrier. Colonic cells can degrade excess BA through an oxidative phosphorylation mechanism, thereby preventing its adverse effects on the colonic mucosa [22]. In contrast, the ileum lacks an intact crypt-protective structure like the colon and cannot secrete sufficient BA-degrading enzymes [22,23]. Therefore, excess BA is more likely to easily reach the ileal crypts and inhibit stem cell proliferation, resulting in IMBD. This finding further confirms the plausibility of this study’s speculations.

Early research reports showed that the intestinal BA content was higher in some patients with mental disorders such as depression and autism [48,49]. More recently, other studies have found BA over-enrichment in the feces of some adolescent animal models of depression and some patients with mental health disorders [50,51,52]. However, these reports have been overlooked because the beneficial effects of BA have attracted much attention. This study demonstrates for the first time a strong association between BA over-enrichment in the ileal lumen and IMBD in adult CUMS model mice. This finding provides a new research direction for the underlying mechanism of CUMS-induced intestinal mucosal injury and, thus, induced diseases such as depression. However, the specific cause of CUMS-induced BA over-enrichment in the ileal lumen has rarely been reported.

Previous reports have correlated BA levels in the gut lumen to the abundance of BA-producing bacteria and the microbiota composition in their ecological niche [53,54]. Despite the abundance of national and international studies on the effects of stress on gut microbiota composition, the results of each study show different characteristics of microbiota composition changes. Significant decreases in the abundance of SCFA-producing bacteria, including *Roseburia* and *Prevotella* [55,56], have been reported. Other studies have revealed that the abundance of SCFA-producing bacteria such as *Faecalibacterium*, *Clostridium*, *Lactobacillus*, and *Bacteroides* was significantly decreased in the feces of the CUMS model group [57,58]. In contrast, the abundance of *Desulfovibrio*, *Streptococcus,* and *Enterococcus* was significantly increased [57,59]. This dysbiosis significantly decreased the levels of SCFAs (especially BA) in the gut [60,61].

In this study, by analyzing the ileal microbiota composition and BA levels in the ileal lumen of the CUMS model mice in three periods, it was found that the ileal microbiota composition and the BA levels in the ileal lumen in different periods are significantly different. A periodic progressive change in the relative abundance of marker ileal bacteria and BA levels under CUMS occurs, with the most striking change occurring at 42 days. The relative abundance of *Lactobacillus*, *Prevotella*, *Turicibacter*, *Blautia*, and *Barnesiella* in the ileum of mice in the CUMS model group is significantly decreased. In contrast, the relative abundance of *Muribaculaceae*, *Butyricicoccus*, *Ruminococcus*, *Roseburia*, and *Eubacterium* significantly increases, and the BA levels increase significantly. *Muribaculaceae*, *Ruminococcus*, *Roseburia*, *Butyricicoccus,* and *Eubacterium* have been reported as SCFA-producing bacteria. In this study, BA levels in the ileal lumen are significantly positively correlated with the abundance of *Muribaculaceae*, *Ruminococcus,* and *Butyricicoccus*. Therefore, it is hypothesized that increased abundance of these three bacteria might be the primary cause of BA over-enrichment in the ileal lumen in the 42 d CUMS model mice. However, the mechanism by which CUMS triggered the massive proliferation of the above BA-producing bacteria still needs to be further investigated.

Previously, it was widely believed that polysaccharides such as dietary fiber were the main raw material for synthesizing SCFAs by SCFA-producing bacteria in the gut [24]. However, recent studies have found that specific AAs, such as glutamate, alanine, glycine, lysine, serine, and valine, can also produce SCFAs through multiple pathways, such as AA fermentation pathway (glutamic acid and lysine), α-ketoacid → acyl-coenzyme A, and γ-aminobutyric acid → butyryl coenzyme A [62,63]. This study shows a significant increase in the gene abundance of aspartate, glutamate, alanine, glycine, threonine and serine degradation, and BA synthesis metabolic pathways in the 42 d CUMS group. Additionally, the FAA levels such as Asp, Glu, Ala, and Gly in the ileal contents are significantly decreased in the 42 d CUMS group compared to the CON group and negatively correlated with MF0089 butyrate production II, MF0088 butyrate production I, and BA (*p* < 0.05). Such data suggest that the AA degradation and BA synthesis metabolism of the ileal microbiota is abnormally hyperactive at 42 d CUMS, inducing the absence of specific FAAs and over-enrichment of BA in the ileal lumen. Therefore, it is hypothesized that the ileal microbiota produces excess BA using specific FAAs.

As gut microbiota is multi-species, a comprehensive understanding of the mechanisms by which the ileal microbiota produces excess BA using specific FAAs at 42 d CUMS requires an understanding of the overall changes in the abundance of critical enzyme genes in the AA degradation metabolism and SCFA synthesis metabolic pathways of the gut flora. Further metabolic prediction and correlation analyses of the ileal microbiota revealed that the gene abundances of AA-degrading enzymes and BA-producing enzymes were significantly increased in the CUMS group (*p* < 0.05) compared to the CON group. The abundance of *Muribaculaceae*, *Ruminococcus*, and *Butyricoccus* significantly positively correlated (*p* < 0.05) with the gene abundance of the specific AA degradation and BA synthesis metabolic pathways and associated enzymes and the BA levels in the ileal contents and significantly negatively correlated (*p* < 0.05) with the Asp, Glu, Ala, and Gly levels. This suggests that ileal microbiota, including *Muribaculaceae*, *Ruminococcus,* and *Butyricicoccus*, produce large amounts of BA. Increased BA levels are of particular note in the presence of specific AA-degrading enzymes through the AA degradation metabolism of FAAs, including Asp, Glu, Ala, and Gly, which act as specific nutrients in 42 d CUMS mice. This study also found that the relative abundance of *Muribaculaceae* significantly increases from 24% in the CON group to 48% in the model group in the 42 d CUMS model group. Such an increase suggests that *Muribaculaceae* is the dominant genus of ileal microbiota in the 42 d CUMS model group and that the magnitude of change was more substantial than other genera. *Muribaculaceae* (formerly known as S24-7) is a core genus in the gut [64], and its members can use FAAs, including Asp, Ala, and Glu, and are involved in the degradation of complex carbohydrates to produce acetic, propionic, and butyric acids [65,66]. Therefore, a significant increase in the abundance of this bacterium would inevitably lead to a significant decrease in FAA levels, including Asp, Ala, and Glu, as well as a significant increase in SCFA levels. Most of the *Muribaculaceae* are challenging to isolate and purify in vitro, due to strict anaerobic requirements, complex nutritional needs, dependency on microbial community interactions, and genomic and metabolic diversity [67,68]. Consequently, there is very little understanding of *Muribaculaceae*, especially their role in CUMS and interactions with other microbiota.

*Ruminococcus* and *Butyricicoccus* have similar effects as *Muribaculaceae*. *Ruminococcus* can produce SCFAs, including butyric, acetic and propionic acids, from FAAs [69,70]. *Butyricicoccus* can produce BA from FAAs [71]. In summary, the abundance of SCFA-producing bacteria (*Muribaculaceae*, *Ruminococcus*, and *Butyricicoccus*) and their specific AA degradation and BA synthesis metabolism, which use FAAs such as Asp, Glu, Ala, and Gly to produce BA, are significantly increased (*p* < 0.05) in the ileal lumen in 42 d CUMS mice, resulting in excessive BA accumulation and a deficiency of specific AAs. BA overaccumulation and specific AA deficiencies can alter the physicochemical environment of the intestinal ecological niche. However, the potential impact of these changes on the abundance of the ileal microbiota is unknown.

Several studies have shown that the different types and concentrations of AA can determine the growth of different types of bacteria, and the AA composition in the luminal ecological niche exerts a critical driving effect on the composition of the gut bacterial community [72]. In addition, increased BA levels in the gut can reduce the abundance of BA-sensitive bacteria through several mechanisms, such as intracellular acidification, metabolic inhibition, oxidative stress and DNA damage, and inhibition of cellular communication [24,73,74,75,76]. In 42 d CUMS model mice, the Asp, Glu, Ala, and Gly levels decreased, BA levels increased, and the abundance of *Prevotella*, *Lactobacillus*, *Turicibacter*, *Blautia*, and *Barnesiella* significantly decreased (*p* < 0.01). Moreover, FAA levels significantly positively correlated with the abundance of the above genera, whereas BA levels were significantly negatively correlated (*p* < 0.05) with their abundance. The genera *Prevotella*, *Lactobacillus*, *Turicibacter*, *Blautia,* and *Barnesiella* widely exist in the gastrointestinal tract of humans and animals [77]. As important AA-fermenting bacteria, the bacteria mentioned above depend on specific AAs for growth [78,79]. Growth of BA-sensitive bacteria such as *Lactobacillus* [80], *Fusobacterium* [81], *Blautia* [82,83], and *Barnesiella* [80] is inhibited by high BA concentrations. High BA concentrations and deficiencies in specific FAAs inevitably significantly reduced the abundance of flora such as *Prevotella*, *Lactobacillus*, *Turicibacter*, *Blautia,* and *Barnesiella*. As the gut microbiota ecosystem is symbiotic with mutual constraints, a decrease in the abundance of one flora type may lead to an increase in the abundance of another type [84,85]. Such an interplay of the two factors was verified by analyzing the abundance of bacteria that rely on specific AAs for growth, including *Prevotella,* which significantly negatively correlated with the abundance of SCFA-producing bacteria (especially BA-producing bacteria), including *Muribaculaceae*. Thus, in the 42 d CUMS model mice, most of the specific FAAs entering the ileal segment were consumed by BA-producing bacteria to produce large amounts of BA. Increasing BA levels and decreasing FAA levels inhibited the growth of BA-sensitive bacteria and/or bacteria that rely on specific AAs for growth, promoting a further enrichment of BA-producing bacteria.

Due to the complexity of the internal environment of organisms, the interaction between FAAs and symbiotic flora in the ileum is regulated by many factors. This study used in vitro culture experiments of ileum microbiota to exclude such interference. It was apparent that after a 12 h culture of the ileal microbiota in the 42 d CUMS model group, the specific AAs in the culture medium were able to replenish the missing AAs in the ileal contents and restore the balance of the flora. However, as the culture time extended, the depletion of specific AAs in the culture medium led to an imbalance in the flora, which resulted in the enrichment of BA-producing bacteria. The in vitro culture experiment successfully reproduced the in vivo results, further supporting the study’s inference.

In summary, this study explored the potential mechanisms through which CUMS induces microbiota-derived BA over-enrichment in the ileum lumen and its correlation with IMBD. The findings provide a novel perspective for identifying potential biomarkers of CUMS-induced IMBD and highlight the possibility of modulating gut microbiota AA metabolism to inhibit excessive BA accumulation. Such outcomes offer not only a promising strategy for preventing and managing CUMS-induced IMBD but also a theoretical foundation for developing functional products targeting this condition.

However, this study has certain limitations. First, it focused exclusively on male mice, leaving uncertainty about whether similar effects would occur in females, though we hypothesize comparable results. Secondly, the gut microbiota–AA metabolism–BA axis involves complex interactions among multiple target microbiota, enzymes, and metabolic pathways. Future research should further elucidate the causes of gut microbiota structural and metabolic abnormalities caused by CUMS through in vitro studies. Thirdly, the impact of CUMS-characterized marker bacteria on BA accumulation will be further validated through ileal microbiota transplantation experiments using germ-free mice. Finally, the propagation and differentiation of ISCs are influenced by numerous factors, and the exact relationship between them and BA enrichment remains unclear and needs further study.

## 4. Materials and Methods

### 4.1. Experimental Animals and Study Design

The experimental conditions for feeding mice were as previously described [86], with some modifications. In total, 90 healthy, 7-week-old, specific pathogen-free (SPF) male C57BL/6 mice (Laboratory Animal License No. SCXK(GD)2020-0051) of average weight (20 ± 2 g) were purchased from Zhuhai Baitantong Biotechnology Co., Ltd., China [batch number SYXK(GD) 2019-0204]. The experimental mice were housed in the SPF-grade animal facility of the College of Food Science and Technology, Guangdong Ocean University, at a temperature of 22 ± 3 °C, a humidity of 60 ± 10% and a 12 h day/light cycle, with free access to feed pellets sterilized with Co60 and distilled water, except when receiving specific treatments during CUMS model establishment. High-pressure steam was employed to sterilize the materials used for the water bottles, cages, and pads. The water bottles and pad fillings were replaced three times each week. Following a week of acclimatization, all 15 mice were each randomly assigned to one of six groups (*n* = 15) [14 d Control (CON 14 d), 14 d CUMS model (CUMS 14 d), 28 d Control (CON 28 d), 28d CUMS model (CUMS 28 d), 42 d Control (CON 42 d), 42 d CUMS model (CUMS 42 d)]. Each group consisting of 15 mice was randomly housed in three cages of 5 mice each.

### 4.2. CUMS Procedure

**Figure 10:**
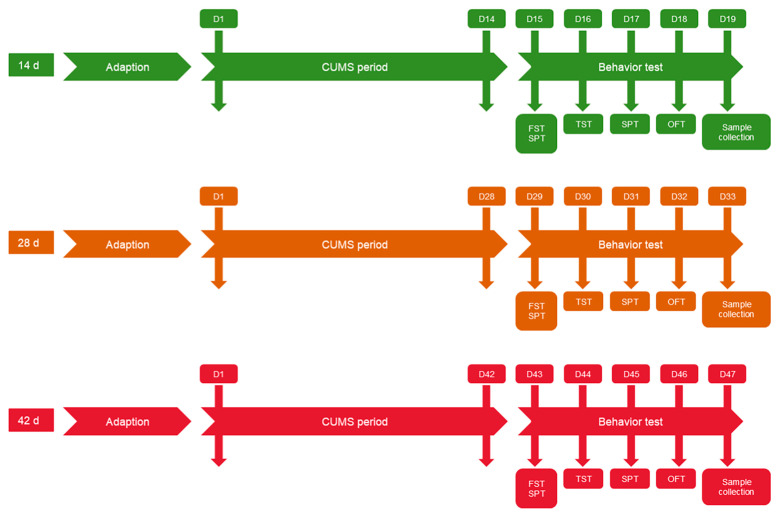
Flowchart of the chronic unpredicted mild stress (CUMS) procedure.

CUMS was conducted according to published methods, with minor modifications [87,88,89,90]. Specifically, 11 mild stressors, 12 h cage tilt (45°), 24 h water deprivation and/or food deprivation, 4 h restraint, 24 h darkness or light, 24 h odor and foreign object stimulation, 15 min tail suspension, 2 h coldness (4–10 °C), 1 h noise (92 dB, 1500 HZ), 24 h moist bedding, 24 h no bedding, and 4 h strobe flash stimulation were conducted. Mice were given two or three of these stressors at a random time per day. No single stressor was applied on 2 consecutive days. CUMS 14 d, CUMS 28 d, and CUMS 42 d groups of mice were subjected to CUMS to simulate an animal model of depression. Three CON (14, 28, and 42 d) groups were left undisturbed. The behavioral assessment tests, including the sucrose preference test (SPT), open field test (OFT), tail suspension test (TST), and forced-swim test (FST) were carried out after each CUMS test period, on days 15, 29, and 43. The mice were sacrificed 24 h after behavioral tests to avoid potential acute effects of the behavioral tests (Figure 10).

### 4.3. Mice Behavioral Experiments

All mice were subjected to behavioral tests to assess the influence of 14, 28, and 42 d CUMS on mice. The SPT, OFT, TST, and FST were conducted as described by [33,91,92,93]. SPT is a behavioral assessment to gauge the emotional well-being of laboratory animals. It serves as a critical metric for determining happiness levels and the extent of depression in mice. In the SPT, the volume of sucrose solution and water consumed by each cage was calculated using the formula, SPT = [volume of sucrose solution consumed/(volume of sucrose solution consumed + volume of water consumed)] × 100%. The OFT is a behavioral method to monitor the intensity of autonomy, inquisitiveness, and alertness of experimental animals in novel and alien environments and serves as a primary measure for evaluating anxiety and fear levels in mice. In the OFT, the cumulative time the mice spent in the center area of an open field (40 × 40 × 40 cm) in a 6 min session is counted. The TST and FST are behavioral tests in experimental animals to assess the intensity of the desire to survive in a desperate environment and represent a crucial metric for determining the severity of depression. The TST measures the immobility time for the last 4 min in the total 6 min of being suspended head down and its limbs not permitted to touch the surrounding objects. The FST measures the duration of immobility time for the last 4 min in the total 6 min of swimming in a transparent, cylindrical glass tank (16 cm diameter and 24 cm high).

### 4.4. Physiological Variables and Sample Collection

Throughout the experiment, the physiological parameters, including body weight and feed intake of the mice, were monitored daily. After 24 h of behavioral experiments, 1 mL of the mice’s blood was collected from the eye, and the mice were euthanized immediately. The blood was centrifuged at 1200× *g*, 4 °C, for 5 min. The serum was collected and stored at −80 °C until required. The organs (heart, liver, spleen, lung, and kidney) were collected, weighed, and the organ index calculated according to the formula: organ index = weight of organ (mg)/body weight (g).

The ileum was immediately collected and divided into two parts. One segment of the ileum was used to collect the ileal contents, which were placed in sterile centrifuge tubes on ice. The ileum and the ileal contents samples were stored separately at −80 °C. The rest of the ileum was immediately preserved in 10% formalin for hematoxylin and eosin (H&E) staining and the immunohistochemistry (IHC) staining.

### 4.5. Measurement of Inflammatory Markers, Diamine Oxidase (DAO), and Lipopolysaccharide (LPS)

The serum inflammatory cytokines, DAO, and LPS levels were measured according to [94], with modifications. The following mouse enzyme-linked immunosorbent assay (ELISA) kits: tumor necrosis factor-α (TNF-α, EMC102a.96), interleukin-10 (IL-10, EMC005.96), interleukin-1β (IL-1β, EMC001b.96), and interleukin-6 (IL-6, EMC004.96), were purchased from Neobioscience Technology Co., Ltd., Shenzhen, China. The DAO kit (G0134W) was purchased from Suzhou Grace Biotechnology Co., Ltd., Suzhou, China. The microplate quantitative chromogenic matrix Limulus kit (EC64405) was purchased from Xiamen Limulus Reagent Biotechnology Co., Ltd., Xiamen, Fujian Province, China. They were used according to the manufacturer’s instructions.

### 4.6. H&E Staining and Analysis of Ileum Tissue

Ileal tissue was prepared and stained based on the method of [94]. Subsequently, 5 μm sections (*n* = 3/animal) were examined under a light microscope to assess histological damage. Measurements were taken for the number of goblet cells, villi length, and crypt depth. Five areas were selected from each sample, and the average calculated for each sample and group. Ileal samples were then histologically scored according to the Chiu’s Small Intestinal Mucosal Injury Scale [95].

### 4.7. Immunohistochemical (IHC) Staining and Analysis of Ileum Tissue

Immunohistochemical (IHC) staining and analysis of ileum tissue were conducted according to the method of [96], with some modifications. The primary antibodies used, namely rabbit anti-ZO-1 (1:1000; Cat No: 21773-1-AP), rabbit anti-occludin (1:2000; Cat No: 27260-1-AP), and rabbit anti-Muc2 (1:2000; Cat No: 27675-1-AP), were purchased from Wuhan Proteintech Biotechnology Co., Ltd., Wuhan, China. Rabbit anti-olfm4 (1:400; Cat No: 39141t) was purchased from Cell Signaling Technology, Inc. (CST), Boston, MA, USA. The ileal tissue slides were washed three times with phosphate-buffered saline for 5 min each. Next, the slides were incubated with ultrasensitive rabbit-mouse universal secondary antibody (Cat No: 39141t; Tuling Hangzhou Biomedical Co., Ltd., Hangzhou, China) for 50 min at room temperature. An automated image analysis software, AIpathwell v2 (Servicebio, Wuhan, China), was then used to analyze the amount of target protein distribution in the ileum.

### 4.8. Gut Microbiota Analysis

Mice ileal contents (*n* = 10) were subjected to 16S rDNA high-throughput sequencing using the Illumina NovaSeq6000 high-throughput sequencing platform (Hangzhou GUHE Information Technology Co., Ltd., Hangzhou, China) [86,94]. Classification annotation was performed by comparing it with the database. The R stats package Kruskal method [97] was used to compare differences in gut flora structure between samples and between groups at each phylum and genus taxonomic level. PICRUSt was used to predict microbial metabolism changes based on the gut microbiota 16S rDNA analysis. The genes determined via macroeconomic prediction were annotated and classified according to metabolic pathways by comparing the protein sequences with those in the Gut Metabolic Module (GMM) databases. The GMM was mapped from the KEGG Orthology (KO) of the Kyoto Encyclopedia of Genes and Genomes (KEGG).

### 4.9. Free Amino Acid (FAA) Analysis

The free amino acid (FAA) analysis was based on the method of [98], with minor modifications. We weighed 0.2 g of ileal contents into a centrifuge tube, added 1 mL of pre-cooled ultrapure water, centrifuged at 4000 r/min for 15 min at 4 °C, and removed the supernatant. The procedure was repeated twice, and the supernatant was collected and filtered through a 0.22 μm aqueous microporous filter membrane. We mixed 100 μL of the filtrate with 100 μL of a phenyl isothiocyanate–acetonitrile solution (0.25:10, *v*/*v*) and triethylamine–acetonitrile solution (1.4:10, *v*/*v*). After standing for 1 h at 20 °C, 300 μL hexane was added and well shaken. The lower part of the solution was filtered through a 0.22 μm organic microporous filter membrane for analysis. The parameters of the liquid chromatograph were set as follows: column temperature = 40 °C, flow rate = 1.0 mL/L, injection volume = 10 μL, detection wavelength = 254 nm. A gradient elution was used with mobile phases of sodium acetate–water–acetonitrile solution (8:900:70, *w*/*v*/*v*) and acetonitrile–water solution (8:2, *v*/*v*). The FAAs of the samples were characterized and quantified by comparing the retention times and peak areas with the 17 amino acid (AA) standards.

### 4.10. Short-Chain Fatty Acid (SCFA) Analysis

The SCFAs in mouse ileal contents were measured as described previously using a gas chromatography–mass spectrometer (GC–MS) [94]. An appropriate amount of six kinds of SCFA standards (analytical grade acetic acid, propionic acid, N-butyric acid (BA), isobutyric acid, isovaleric acid, and N-valeric acid) was purchased from Shanghai Macklin Reagent Biotechnology Co. and was weighed and dissolved in diethyl ether to create fourteen mixtures of standard concentration gradients. The samples were thawed on ice to extract polar metabolites for the GC-MS analysis. A total of 100 µL of 15% phosphoric acid was combined with around 50 mg of each sample. Then, 100 µL of an internal standard solution containing 125 µg/mL of isohexanoic acid from Shanghai Aladdin Biochemical Technology Co., Ltd. was homogenized for 1 min with 900 µL of diethyl ether. The material was then centrifuged for 10 min at 12,000× *g*, 4 °C. The combined solution was filtered using an organic microporous membrane with a 0.22 µm pore size, and the supernatant was then collected and examined using a GCMS-TQ8050 NX device. GC condition: Capillary column (0.25 mm × 30 m, 0.25 µm, Agilent, VF-17MS). The injected sample volume for the GC-MS analysis was 1 μL with a split injection ratio of 5:1. The injection port temperature was 250 °C, and the temperature of the FID was 230 °C. The carrier gas was helium, with a carrier gas flow rate of 1 mL/min. After starting at 90°C, the column’s temperature was raised by 10 °C/min to 120 °C, kept for 1 min, then increased by 5 °C/min to 150 °C, again sustained for 5 min, and then raised by 25 °C/min to 250 °C. The electron impact (EI) ion source and a source ionization energy of 70 eV were the MS conditions, and SIM was used to gather the data. The quantitative measurement of fecal SCFAs was carried out using the calibration curve method. Peak-area integration was accessed based on the retention time.

### 4.11. Ileal Flora In Vitro Culture

The ileal flora in vitro culture was performed based on the in vitro fermentation method of [99]. Briefly, the ileal contents of CON 42 d and CUMS 42 d mice were collected and added to the Gifu Anaerobic Medium (GAM) growth medium (pH 7.2) to prepare the ileal mixture (10%, *w*/*v*). The mixture was homogenized for 1 min, centrifuged (500× *g*, 4 °C, and 5 min), and the supernatant, the ileal slurry, was immediately transferred into an anaerobic jar. The ileal slurry was inoculated into the GAM growth medium at 1% inoculum and incubated anaerobically at 37 °C. The fermentation broths of the colonies were collected every 6 h, and their absorbance was determined at a wavelength of 600 nm (OD_600_) for 30 h to plot the microbial growth and proliferation profile of the ileal slurry. The fermentation broths of the colonies were collected at 12 h (12 h CON 42 d, 12 h CUMS 42 d) and 24 h (24 h CON 42 d, 24 h CUMS 42 d), respectively, and their pH, FAA, and BA levels, and microbial composition and function were determined (*n* = 6).

### 4.12. Statistical Analysis

All results are presented as mean ± standard error of the mean (SEM). SPSS 21.0 (SPSS, Chicago, IL, USA) was used for statistical analysis. Independent sample *t*-test was used to analyze the significance, with *p* < 0.05 regarded as statistically significant. The data were plotted using Origin 2022 software. Pearson’s method was used to analyze data correlation.

## 5. Conclusions

In conclusion, this study reveals that with the length of time (14, 28, and 42 d) of exposure to chronic unpredictable mild stress (CUMS), the ileal mucosal barrier damage (IMBD) progressively worsens. In the late CUMS stage (42 d), the abundance of bacteria (*Muribaculaceae*, *Ruminococcus*, and *Butyricicoccus*) that produced BA by using specific free amino acids (FAAs), such as aspartic acid (Asp), glutamic acid (Glu), alanine (Ala), and glycine (Gly) in the ileal lumen significantly increases, resulting in excessive accumulation of BA and deficiency of specific AAs in the ileal lumen. Such an imbalance inhibits BA-sensitive bacteria proliferation and other bacteria that rely on specific AAs for growth (namely, *Prevotella*, *Lactobacillus*, *Turicibacter*, *Blautia*, and *Barnesiella*), thereby reducing their relative abundance. These changes, in turn, promote further colonization of BA-producing bacteria, exacerbating the over-accumulation of BA in the ileal lumen, thereby inhibiting the proliferation of intestinal stem cells (ISCs), ultimately leading to IMBD.

## Data Availability

Raw sequence reads were deposited in the National Center for Biotechnology Information sequence read archive, Bioproject PRJNA1168879. Further data are available from the corresponding author on reasonable request.
